# Perceptions of the English Use of College Transfer Nursing Students in a Non-English Speaking City: A Qualitative Study

**DOI:** 10.3390/ijerph17020462

**Published:** 2020-01-10

**Authors:** Shirley Siu Yin Ching, Dennis Foung, Lillian Weiwei Zhang, Gwendoline Yuanyuan Guan, Kin Cheung

**Affiliations:** 1 School of Nursing, The Hong Kong Polytechnic University, Yuk Choi Road, Hung Hom, Kowloon, Hong Kong, China; shirley.ching@polyu.edu.hk (S.S.Y.C.); lillian.zhang@polyu.edu.hk (L.W.Z.); gwendoline.guan@polyu.edu.hk (G.Y.G.); 2 English Learning Centre, The Hong Kong Polytechnic University, Yuk Choi Road, Hung Hom, Kowloon, Hong Kong, China; dennis.foung@gmail.com

**Keywords:** nursing, use of English, motivation, college transfer students, non-native English speakers, qualitative study, cultural context

## Abstract

There has been limited research on nursing students’ (NSs) language problems conducted in non-English speaking countries, especially research focusing on college transfer students. The purpose of this study was to explore the perceived needs and challenges of English use by college transfer NSs in a non-English speaking environment. A descriptive study design was adopted. Forty-five college transfer NSs from a university in Hong Kong participated in the study. Sixteen interviews were conducted. A qualitative content analysis was performed. Two main categories were identified: (a) Perceptions about English use (three sub-categories: (i) significance of having a good command of English; (ii) challenges in use of English; and (iii) low motivation and need to learn English), and (b) using English in nursing contexts (two sub-categories: (i) challenges in the use of English in nursing and (ii) improving English proficiency as a second priority in nursing students). In conclusion, college transfer NSs face challenges in general and discipline-specific English use, but their motivation to improve their English proficiency was not strong. Language centers should re-design the language courses to meet NSs’ communication needs, while nursing educators should provide opportunities for students to strengthen their English use in research and clinical situations.

## 1. Introduction

English has long been considered a lingua franca, being used for communication between people with different mother-tongues [1]. Competence in English impacts a variety of professionals, particularly nurses [2]. In fact, there is an increasing need for the use of English across non-English speaking countries. For example, people from English-speaking countries may travel to Asia for medical services [3]. The lack of a guarantee of English communication might hinder the country from providing excellent healthcare to diverse populations [4]. Therefore, a growing need for effective English communication for medical purposes is evident in non-English speaking countries around the globe.

Communication in healthcare can take many forms [5,6]. Lu [2], in her study in Taiwan, described two ways in which healthcare professionals need to use English: (1) using medical jargon with medical professionals and (2) using daily-life English with patients from foreign countries, to explain nursing procedures or to calm them down. Lu [2] gave a clear explanation of the function of communication, but the ways in which language is used have not yet been given sufficient consideration. For example, through any kind of communication, healthcare professionals have to show empathy and compassion [7]. While the function of communication can be direct, the subtlety and tactfulness in healthcare communication are rather sophisticated.

As the languages that nurses can use depend on the languages they learn in nursing schools, studies have been conducted to understand the language problems they face [8,9,10]. English is a critical subject for nursing students (NSs) because, apart from its uses in the workplace, it is the major language used to communicate in top-tier medical research papers [11]. Wang, Singh, Bird and Ives [12] found that NSs who are non-native English speakers (NNES) might have problems with oral communication, reading, writing, and even their awareness of cultural issues. In a general academic context, Johnson [8] and Starkey [10] reported that NNES NSs encounter problems understanding lectures; even verb tenses can be problematic for them [9]. Without a doubt, English proficiency can facilitate a nurse’s mobility. In a global mobility study on nurses, China (including Taiwan and Hong Kong) ranked in the top 10 in terms of nurses taking the Commission on Graduates of Foreign Nursing Schools (CGFNS) Exam (1990–2006), which made English learning essential for professional development [13]. Unfortunately, the above review may not have represented the whole picture of NNES NSs, as almost all studies were conducted in English-speaking countries, i.e., an NNES as an NS in an English-speaking country. Very few studies have been conducted on the language needs of NNES NSs in a setting where English is a second or foreign language. Among the few investigations that have been completed, Thai NSs experienced anxiety in the use of English [14]. The young generation in Hong Kong were also found to have low motivation in terms of learning English [15]. Therefore, there is an urgent need to understand the language needs of NNES NSs in non-English speaking countries.

While language issues are relevant for NSs in different education programmes, the language needs of college transfer NSs have been ignored. College transfer students are those who have completed a two-year post-secondary qualification and have transferred to a 4-year university bachelor degree programme [16]. From an international perspective, many nurses who only have post-secondary-school qualifications find it necessary to meet the rising levels of qualifications in the profession [17]. From an institutional level, nursing college transfer students have attracted the attention of the academic community, and many studies have attempted to help them in various contexts by exploring the critical success factors for this group [18]. An effective transfer will lead to improved academic performance, but failure may lead to students dropping out of programmes [19]. Some critical language issues for college transfer students include the need to handle greater volumes of readings and higher expectations to perform at the levels required by scholarly writing [20]. Therefore, it is vital to examine the language needs of college transfer NSs.

The current study was conducted in Hong Kong, typical of the contexts described above, where the training of NNES NSs happens in a non-English speaking city. Even though most people in Hong Kong speak Cantonese, the language that is the second most popular is English [21]. It was previously a British colony and returned to China in 1997. Both Chinese and English are the official languages [22]. Traditionally, nursing education here follows the British system [23], whereby English is used for formal documentation in the health care system. Because of its unique history, English is the medium of instruction in nurse education, universities, community colleges, and most high schools. High school graduates are admitted to university studies based on the results of the Hong Kong Diploma of Secondary Education (DSE). The Nursing Council of Hong Kong (NCHK) has stipulated the minimum requirement of English language of level 3 or above (ranging from unclassified to level 5) as one of the minimum entry requirements for nursing education [24]. Normally, those whose marks are not high enough to gain admission to university studies might consider studying sub-degree programmes (graduating with an associate degree or a higher diploma) in community colleges. Compared with students of other non-health disciplines, the challenges NSs face are two-fold [25]. On the one hand, they have to learn the language of health sciences; on the other hand, they have to fulfill the high academic requirements of the discipline. College transfer students are an essential source of NS candidates worldwide [19,26], including in Hong Kong. Many of them are admitted to universities through credit transfer or exemption, including the English course. However, how they use English to meet the rigorous requirements of both academic studies and professional preparation is unknown. It is critical to investigate their needs and use of English in their post-transfer studies at four-year degree universities.

To understand the language needs and challenges faced by college transfer NSs, our two specific research questions were

1. How do college transfer students perceive the challenges and needs of having a good command of English?
2. What challenges do college transfer students face when using English in a nursing context?

## 2. Methods

### 2.1. Design

Perception of use of English in higher education is a phenomenon that is affected by dynamic personal and situational factors. A mix of individual and focus group interviews were conducted to allow the participants to discuss complex subjects in an amenable setting, and permit them to reveal what they think and why they think what they do. The burden of high-effort cognitive thoughts was shared among the members in the group. Through the interaction and discussion, they could work together to tackle complicated ideas and concepts [27]. Qualitative content analysis as stipulated by Graneheim and Lundman [28] was conducted because it allowed both a broad and an in-depth understanding of the phenomenon to be obtained [29].

### 2.2. Participants and Setting

The participants were recruited through purposive sampling from a university in Hong Kong. Selection criteria included (a) college transfer NSs who graduated from a two-year community college and studied in a three-year bachelor programme leading to nursing registration or their graduates; (b) those who had been studying in the programme for at least one semester; (c) Chinese ethnicity; and (d) speaking English as a second language (i.e., use Chinese in their daily life, but English in a wide range of contexts in their daily life). Invitation emails were sent to all college transfer NSs and graduates (who had graduated within one or two years) in the three-year bachelor programme. The participants who replied to the email and met the selection criteria were invited to join interviews according to their years of study (i.e., year 1, 2, or 3). Target NSs were also invited in class or through faculties (who would not know students’ participation status). There was no prior review of the students’ personal information before recruitment. Interviews were conducted in a quiet room at the research site.

### 2.3. Data Collection

In total, 12 focus group and 4 individual interviews were conducted with 45 NSs (9 male and 36 female) from February to July 2018. The numbers of year 1, 2, and 3 students and graduates were 14, 13, 9, and 9 respectively. Depending on the students’ availability, each interview consisted of 1–5 participants, with an average of three students per group. The interviews lasted for 30–45 min. Each student was interviewed once, based on the interview guide (Table 1). The interviewer was a female research assistant with experience in qualitative studies and was not acquainted with the students. She met the participants on the day of the interview, and explained the purpose, procedures, and principles of voluntary participation and confidentiality. Each participant was given an information sheet and opportunity to clarify their queries before signing a consent form to participate in the study.

A focus group approach is a method of collecting data on a designated topic by using a semi-structured group session that is moderated by an interviewer in an informal setting [30]. During the interview, the interviewer should be active in creating a group discussion for data collection [31]. A funnel-based approach was adopted [32]. The interviews started with “Can you tell me about your experience of using English, till now, in the university?” Depending on the answers, probing questions were asked, which allowed the participants to elaborate on their experiences of their English use and challenges encountered. The interviewer explored the diversity of experiences among groups of students and compared the categories that were identified from the previous interviews. All the interviews were conducted in Cantonese. At least one research assistant joined each focus group and recorded the interactions among the participants. Debriefing sessions were held between the interviewer and the research assistant after each session to note key themes, hunches, and interpretations. Data collection and data analysis occurred simultaneously. The interviewer and research assistant were involved in both processes. The data collection continued until data saturation, when no additional new categories or important information could be identified in the data from the next few interviews [33].

### 2.4. Data Analysis

The interviews with NSs were analyzed using manifest qualitative content analysis [29]. The audio-recordings of the interviews were transcribed verbatim in Chinese and then imported into NVivo Pro 12 for data management and analysis. According to Graneheim and Lundman [28], qualitative content analysis consists of five steps. First, the researchers read and re-read the interview transcriptions to get immersed in their data pools. Second, using an inductive approach, meaning units (sentences/paragraphs) corresponding to different aspects of the students’ experiences (e.g., “learn English well”), the challenges (e.g., “fail to express ideas in writing”) and needs (e.g., “need writing support in Final Year Project”) of learning English were selected. Third, every meaning unit was condensed and labeled with codes. For example, experience (e.g., “good command of English is important for study”), challenges (e.g., “difficulties about writing in English”), and needs (e.g., “need to learn English is low”). Fourth, subcategories were identified and grouped by comparing their similarities and differences. Finally, two categories (e.g., “perceptions about English” and “using English in nursing context”) were identified.

The interviewer and research assistant coded four interviews independently. The intercoder reliability was checked manually. Then, they discussed their codings to ensure consistency in coding and agreement on a set of codes and categories for further analysis of the subsequent interviews. The trustworthiness of the study was ensured. Peer debriefings about the data coding were done by the interviewer, the research assistant, and the team members, to ensure credibility. Transferability was maintained by providing contextual details and rich descriptions of the findings. Dependability and conformability of the findings were ensured by keeping an audit trail [34].

### 2.5. Ethical Approval

Ethical approval was obtained from the Human Subjects Ethics Sub-Committee of the Hong Kong Polytechnic University (HSEARS20170808003).

## 3. Results

The objective of this study was to explore the perceived challenges and needs of college transfer NSs in terms of having a good command of English and using English in a nursing context. Two main categories were identified: “Perceptions about English” and “Using English in the nursing context” (Table 2).

### 3.1. Perceptions about English

#### 3.1.1. Significance of Having a Good Command of English

All participants had been admitted to community colleges after completing their high school studies. English was a core subject in their high schools and community college studies. They were required to write assignments and present their work in English. They believed that their ability to use it was important to their studies and career development:

“English is important. Everything was written in English, except those (topics) on Chinese medicine…. It is also important when working in the hospital.”.

(24A)

“Even though we do not use too much English in the workplace, we need to learn English well. …we may pursue a master’s degree or work in a foreign country in future. I think making a solid basis of English is very important.”.

(24B)

#### 3.1.2. Challenges in the Use of English

Students valued the significance of English in their study. At the same time, they noted their inadequacy in the use of English. The difference in the sentence structure between English and Chinese exacerbated the problem. Some students said,

“Yes. There is a lot to improve regarding fluency or accuracy in use of English.”.

(05E)

“I am weak in listening. I am fine in lectures…But when I travelled to other countries, I couldn’t understand the foreign language because of the people’s accents and speed.”.

(01A5)

“I don’t know how to express my ideas in writing, which may be related to sentence structure.”.

(14A)

Furthermore, some NSs had not developed the habit of thinking in English and their need to translate between Chinese and English resulted in delays in communication and difficulties in following lectures. They said that they might even respond in Chinese when the teacher asked them questions in English:

“If I was asked to express all my thoughts, it would take me a little longer time to think. I do not think in English. …I am shy to speak English.”.

(05A3)

“I had to load the message when the teacher spoke too fast, I was not quick enough to follow. I may have missed something.”.

(07D)

The difficulty in listening, reading, speaking, and writing in English hindered learning among the transfer nursing students.

#### 3.1.3. Low Motivation and Need to Learn English

It is logical to expect that the students who acknowledged the significance of a good command of English and their inadequacy would be highly motivated to improve their English. However, the need of NSs’ to learn and improve English was not high in general. The students believed that they were capable of studying nursing in the English medium, despite the inadequacies they identified in their use of it. Moreover, studying nursing required them to use English in a specific way that was different from what they learnt in high school. The exclusion of English as a core subject in university lowered their motivation to learn English:

“I think I can handle the (nursing) study.”.

(08A2)

“You should improve English before DSE. If you passed DSE, why should you improve your English?”.

(05A2)

“Learning English was compulsory in the sub-degree…In the university, the motivation is for practical use in Nursing.”.

(14B)

### 3.2. Using English in the Nursing Context

#### 3.2.1. Challenges in the Use of English in Nursing

Almost all students said they had found it difficult to adjust to the use of English in teaching and learning in their community colleges and universities at the beginning of their nursing studies. Those who came from Chinese-medium high schools encountered more challenges because they needed to catch up with the use of English for learning:

“I graduated from a Chinese secondary school. I had English language subjects in my secondary school, but all the other subjects were conducted in Chinese… I had to catch up by myself, such as checking a dictionary. Finally, I am fine with listening to lectures in English now.”.

(21B)

“I attended an English-medium secondary school… I started to read nursing papers written in English when I was studying in the sub-degree programme. I checked the dictionary for unfamiliar nursing terminology. When I entered university, I could read nursing papers quickly.”.

(21D)

In addition to adjusting to learning in English, some NSs faced specific challenges in understanding the nursing subject content during lectures, because of unfamiliar medical vocabulary, medical prefixes, suffixes, and abbreviations:

“We had courses, in the sub-degree, focusing on prefixes…suffixes...medical terms. I didn’t know how to apply them. When I studied medical terms, I had to memorize (instead of understand) them …I don’t think it is a good way.”.

(09B)

“… too much vocabulary, you have to make your own effort.”.

(07AB)

“I can’t spell the medical terms even if my teacher reads and explains them in the lecture.”.

(07AB)

Besides, NSs were required to learn academic writing and nursing research skills, which heavily depended on their English competence. A student elaborated on the difficulties she encountered as follows:

“I cannot understand the content of the literature due to both subject matter and use of the English. We don’t know much about how to read journal papers… then it is so hard for us.”.

(13ACD)

Another challenge is the documentation in the patients’ hospital record. When the NSs had clinical placements, they communicated with their patients and other healthcare professionals in Cantonese (i.e., spoken Chinese), but their documentation was in English. Documentation in clinical settings requires other specific uses of English. Some students said,

“The ‘English’ used in nursing is not English. We use abbreviations and the people (nursing staff/students) can understand it. It is not a proper formal language.”.

(05A2)

“We need to write patients’ notes and progress in English. Use of appropriate terms is enough, accuracy in grammar is not required.”.

(02A1)

Students experienced difficulties in learning medical terms and academic writing. They found that the accurate use of medical terms was essential, rather than English usage, because there was a limited use of English in clinical settings.

#### 3.2.2. Improving English Proficiency as a Second Priority in Nursing Students

Transfer students do not need to take English subjects in the university, because their prior learning in the community college has been recognized. The majority of participants were not very keen to improve their English skills. Moreover, compared with nursing subjects, the grades of which contribute to the final graduation award, improving English was not a priority for some NSs. Some said,

“We don’t have an English subject now at university. Now I do not have an objective to achieve good English.”.

(05A2)

“I don’t have confidence. I am afraid that I can’t improve my English with the time invested. The cost of improving English may be the overall GPA.”.

(05A3)

The competing schedules of nursing-specific subjects and general studies further deter NSs from improving their English. One student said,

“I have many things to do and deadlines to meet; assignments, projects, tests… I do not have spare time to focus on English.”.

(08A2)

Without the need to take an English course in the university, there was room for the students to choose their own ways of improving their English. Intrinsic motivation of the students became a determining factor for them to engage in strategies to improve English. Two students said,

“It can enhance my confidence that if I can respond to Professor’s questions or make good group presentation in English. If I can speak excellent English among peers, it is a kind of self-affirmation.”.

(09B)

“Yes, I did before (following intrinsic motivation). I attended English Learning Centre (ELC) speaking class each week. Teacher would give feedback on classroom discussion or teach vocabulary.”.

(01A1)

In addition to using formal resources provided to them in the University (e.g., ELC), some students use informal sources:

“There are many non-local students living in the student hall. I practice speaking (English) with them a lot. I think I can improve by speaking more.”.

(14B)

“I would watch more YouTube videos. I found them very interesting, and I can learn English especially communication skills as well as grammar.”.

(13C)

Without the requirement to study English as part of the curriculum, the students put more emphasis on nursing subjects that carried assessment requirements. Competition for the students’ time and energy among tests, assignments, and clinical placements further reduced their motivation to improve English. Students with intrinsic motivation found ways to improve their English.

## 4. Discussion

Our study is one of the pioneers in exploring English use among college transfer NSs from a non-English speaking place in an Asian country. Our qualitative results indicate that NSs perceived English as necessary in their academic studies, but not so much for career purposes.

### 4.1. Perceived Needs for English

Other than academic purposes, most NSs could only find a limited use of English for their career purposes. Similar to past studies in Asian countries, after their placement experiences, they know that they need to use medical jargon [2], and they think that they are competent enough to use “daily English” to communicate with their foreign patients. This perception might be due to the fact that the majority of clinical placements are in public hospitals, where most of the patients are local Chinese. Nurses might not even have time to communicate with local patients [35] because of the severe shortage of nurses and consequent high nurse-patient ratios [36]. Therefore, the hectic working environment might not nurture NSs’ understanding of how complicated communication can be when they have to communicate with a patient in English, in a tactful manner to show compassion [7]. In general, those who travel to Hong Kong for medical services will be admitted to private hospitals [4]. It has been observed that more nurses are choosing to work in private hospitals and will therefore be likely to encounter such patients [37], but it seems that NSs might not foresee the need to improve their English for potential careers in the private sector. As a result, they only see their needs from an academic perspective, and not from a career perspective.

### 4.2. Challenges in Using English in a Nursing Context

The challenges with using English in nursing studies reported by the students in our study are consistent with findings presented in previous research, but in an unexpected manner. Past studies conducted in English-speaking countries have reported language-learning problems in reading, listening, and grammar [8,9,10]. The students in our study reported similar problems in language use, even though they were completing a degree in a non-English speaking city. This unexpected consistency can be explained by the intake of students. The profile of the students in our study, i.e., undergraduate students, is comparable to that of international students who have just entered university [38]. Therefore, it is possible that both groups of students are encountering similar problems with language learning, but these might be at different levels. Our NSs stated that their assignments were marked based on the subject matter instead of the use of English. However, the inappropriate use of English might not be accepted by English-speaking nursing teachers in English-speaking countries.

In addition, past studies have often reported a lack of cultural awareness to be a problem related to language [39] in English-speaking countries. However, this was not revealed as a problem by the students in our study. Once again, this can be explained by the students’ lack of authentic experiences in dealing with foreign patients. Without such intercultural experiences, they do not see the real need for cultural awareness.

### 4.3. Coping with Language Challenges—A Second-Tier Issue

Another noteworthy issue is the motivation for language learning described by this group of students. Previous studies in English-speaking countries have revealed that the NNES NSs do work hard to cope with the increasing need for English [40], and nurses in these countries have to attain minimum English standards [41]. However, in our study, the students in the context of Hong Kong, where English is not the first language, perceived it as an entry requirement for university study only, and were not motivated to work hard at learning it further. This low motivation can be explained by the fact that, as they revealed, English is not assessed in their nursing assignments. Therefore, they did not pay much attention to the use of English and focused their energies on other elements of their assignments to achieve better results. Such assessment-oriented approaches are not uncommon in university students [42].

However, the low motivation for language learning is a worrying sign for nursing educators, as students in both non-English speaking and English-speaking countries have to face English-speaking patients and connect with an academic world in English. Perhaps nursing educators can better motivate NSs to study English by including correct usage as one of the criteria in the assessment rubric. Furthermore, our results found that research skills were new to the students. Perhaps students can be motivated to learn English by enhancing their research skills. This can be done by introducing students to World Health Organization’s International Classifications of Nursing Diagnoses and Interventions [43]. This can serve as a glossary for students to learn a wide range of nursing terminologies in research and NSs will find this motivating because of its relevance. On the other hand, nursing curricula might involve NSs interacting with native English speakers not only in learning contexts, but also in authentic clinical contexts, such as overseas exchange programmes.

### 4.4. Language Needs—Interplay of Various Factors

Meeting students’ language needs can be a role for language centers in universities. How language courses can meet the needs of students in different disciplines has long been a topic of debate between discipline leaders and English teaching professionals. Language professionals have suggested that writing skills and planning for assignments are a key requirement for NNES students [44], and language courses are designed with a strong focus on writing (as echoed by the students in our study). However, these writing courses do not seem to meet the career needs of NSs who will be required to interact with patients in conversational English and with professional staff using medical jargon [2]. This can be considered as a gap to be addressed by language professionals and more communication-based courses can be designed to meet the needs of the NSs.

### 4.5. Limitations

Because of the participants’ availability, four of the interviews were conducted with individual participants rather than in a focus group, which might have affected the dynamics of discussion and elicitation of ideas. Moreover, some participants may not have felt comfortable discussing personal concerns, such as poor academic results. However, since the data reached saturation after we conducted 16 sessions of interviews, the trustworthiness of our findings should not have been affected by these individual interviews.

## 5. Conclusions

This study has identified students’ perceptions of the challenges they face with the use of English. The results show a clear need to conduct further studies on this topic, particularly for NSs in non-English speaking countries, in addition to the existing research being conducted in English-speaking countries. Both groups of students described similar challenges in their studies during their undergraduate journeys, but their perceptions differed with regard to the use of English in their careers. These NSs did not see a practical need to use English in a non-English speaking environment and seemed to underestimate the sophistication needed in nursing communication. Therefore, they did not have the strong motivation to improve their English that their counterparts in English-speaking countries have. Due to these perceptions and needs, language centers in universities may need to re-design the writing-focused courses for NSs to improve their English in research and communicate with patients in spoken language. Nursing educators can perhaps provide opportunities for NSs to deal with clinical situations in English. These may motivate NSs to see the need for English in their careers and improve their English proficiency.

## Figures and Tables

**Table 1:** Interview guide for exploring the perceived needs and challenges of the English use of college transfer students.

General Broad Opening Question	
1.	Can you tell me about your experience on use of English, till now, in the university?
**Probing Questions**	
1.	When considering your English proficiency as a whole, which aspects are you good at in terms of English? Which aspects are you weak at in terms of English?
2.	In terms of the use of English, which area do you think you need help with?
3.	Have you experienced any problems in learning or use of English?
4.	Have you experienced any problems in studying nursing that is related to English?

**Table 2:** Summary of categories and sub-categories.

Categories	Sub-Categories
Perceptions about English	(i) Significance of having a good command of English
	(ii) Challenges in use of English
	(iii) Low motivation and need to learn English
Using English in nursing context	(i) Challenges in the use of English in nursing
	(ii) Improving English proficiency as a second priority in nursing students
